# Knowledge, attitudes, and practices of pregnant Jordanian women towards physical activity in pregnancy: A cross-sectional study

**DOI:** 10.1097/MD.0000000000042149

**Published:** 2025-04-11

**Authors:** Ahlam J. Alhemedi, Othman Beni Yonis, Nour Abdo, Haya Ali Salem, Esra’a Alomari, Risan Fahmi Alrosan, Qutaiba Alfaqeh, Emran Musadaq Hamza, Abdallah Y. Naser

**Affiliations:** a Department of Public Health and Family Medicine, Faculty of Medicine, Jordan University of Science and Technology, Irbid, Jordan; b Department Obestatric and Gynaecology, Kasr Al Ainy Medical School, University of Cairo, Cairo, Egypt; c Department of Applied Pharmaceutical Sciences and Clinical Pharmacy, Faculty of Pharmacy, Isra University, Amman, Jordan.

**Keywords:** attitudes, knowledge, physical activity, practices, pregnancy

## Abstract

Being inactive before becoming pregnant increases the risk of not initiating exercise during pregnancy. Although exercising has numerous advantages and physical inactivity can be detrimental, research indicates that the majority of pregnant women do not engage in regular exercise. This study aimed to examine knowledge, attitudes, and practices of pregnant Jordanian women towards physical activity during pregnancy. This is a cross-sectional observational study that was conducted between November 2022 and June 2023 in Irbid, Jordan. Jordanian pregnant women were interviewed using the survey tool with the assistance of doctors in the participating health centers. The questionnaire tool examined physical activities profile, knowledge of safety of practicing specific physical activities regularly during a healthy pregnancy, and perception, motivations and barriers related to physical activities during pregnancy. A total of 429 participants were involved in this study. Around 69.0% of the study participants reported that they practiced moderate intensity physical activities outside pregnancy during the past year and only 42.0% achieved the recommended duration of 2.5 hours per week. More than half of the study participants (64.8%) reported that they practice moderate physical activity during pregnancy but only 28.9% achieve the recommended duration of 2.5 hours per week. The mean knowledge score among the study participants was 1.8 (SD: 0.8) out of 5 (represents 36.0% of the maximum attainable score); which reflects low level of knowledge of physical activity during pregnancy. Binary logistic regression analysis identified that higher education level and working in the medical field were factors that are associated with higher likelihood of being knowledgeable of physical activities during pregnancy (*P* < .05). Older participants (aged 31.6 years and above) were 70.0% more likely to practice physical activity during pregnancy compared to others (*P* < .01). This study found disparity in reported physical activity levels, and inability to reach specified exercise duration indicates the need for targeted interventions. The impact of age, smoking status, education, and occupation on knowledge and practice implies that different subgroups need different methods. Next studies should focus on creating and implementing effective educational programs and interventions to promote health-conscious physical activity during pregnancy.

## 1. Introduction

Many guidelines have acknowledged and thoroughly discussed the significance of engaging in physical exercise during uncomplicated pregnancy. The American College of Obstetricians and Gynecologists advise that pregnant women, without any difficulties, should participate in moderate-intensity physical exercise for a minimum of 150 minutes each week. Besides, it is preferable to spread this activity among many days of the week.^[1]^ Physical activities such as brisk walking, cycling, swimming, dancing, and Yoga are suitable for pregnant women to engage in during pregnancy.^[2]^

Research has indicated that engaging in moderate intensity physical activity for the recommended duration during pregnancy does not lead to miscarriages or fetal death. Furthermore, it does not pose any harm to the baby or the mother. On the contrary, pregnant women who engage in physical activity during pregnancy have a reduced risk of developing pre-eclampsia, gestational diabetes, gestational hypertension, urinary incontinence, excessive weight gain, and depression.^[2]^ This is of a special importance as previous research indicated high level of psychological illnesses such as anxiety and depression in the Middle east region including Jordan.^[3–6]^ In Jordan, the overall prevalence of physical activity among the general population is low, similar to other Arabic countries. A cross-sectional study conducted in 2015 on 3196 individuals in Jordan revealed that only 12.5% of the participants engaged in regular physical activity. Furthermore, males exhibited higher levels of physical activity compared to females.^[2]^ There was just one study conducted on the practice of physical activity in pregnant women in Jordan, and the findings indicated a low level of engagement.^[7]^

A study demonstrated that women who failed to meet the recommended level of physical activity during the later stages of pregnancy experienced more weight gain throughout pregnancy compared to women who met the recommendations.^[8]^ Excessive gestational weight gain can lead to health issues for both the mother and baby, including increased risk of maternal and fetal death, gestational diabetes mellitus, and the need for cesarean sections.^[9]^ Women who have substantial weight gain during pregnancy also tend to retain more weight after giving birth, which in turn increases the likelihood of obesity following pregnancy.^[10,11]^ Prior research has established that being inactive before becoming pregnant increases the risk of not initiating exercise during pregnancy. This confirms that women who are already in the habit of exercising before pregnancy are more likely to continue doing so, while those who were not physically active before pregnancy are unlikely to start exercising during pregnancy.^[12]^

Although exercising has numerous advantages and physical inactivity can be detrimental, research indicates that the majority of pregnant women do not engage in regular exercise, and less than 20% adhere to the recommended exercise requirements.^[8,12]^ A study conducted on a sample of 200 pregnant women in India aimed to evaluate their knowledge and attitudes towards prenatal exercising. The findings revealed that 71.4% of the participants reported a lack of adequate information regarding antenatal workouts. Given that this was the primary obstacle, it is possible that enhancing knowledge about the advantages of exercise by educational initiatives and providing appropriate guidance during prenatal visits could enhance the result. The primary obstacles to exercising were fatigue (69.4%) and fear of physical activity (67.3%), as shown in a longitudinal study of Australian pregnant women.^[13]^ Nevertheless, there is a noticeable disparity between knowledge and action, as just 18% of women who possessed knowledge about exercises really engaged in physical activity, despite approximately 50% of them comprehending the associated benefits. The financial cost of implementing exercise as an intervention is minimal, however the impact it has on the health and well-being of both the mother and fetus is substantial. Previous studies in the Middle east and Jordan examined knowledge, attitudes, and practices towards different diseases and health conditions,^[14–17]^ however, none of them examined pregnant women physical activity profile. The aim of this study was to examine knowledge, attitudes, and practices of pregnant Jordanian women towards physical activity during pregnancy.

## 2. Methods

### 2.1. Study design and settings

This is a cross-sectional observational study that was conducted between November 2022 and June 2023 in Irbid, Jordan. Jordanian pregnant women were interviewed using the survey tool with the assistance of doctors in the participating health centers. The utilization of self-administered questionnaire during the face-to-face interview eliminated the possibility of having missing data and all participants completed every item of the survey. Besides, this approach enabled clarification of any misunderstanding and reduced the possibility of response bias. All participants were provided with information about the study before beginning the survey and informed of their option to withdraw at any time. In addition, written informed consent was obtained from all participants.

### 2.2. Study population

The inclusion criteria for this study were to be pregnant Jordanian women or previously pregnant women within the reproductive age group (18–45 years). Pregnant Jordanian women outside the age range < 18 or > 45 years or those who never got pregnant were excluded.

### 2.3. Questionnaire tool

A preliminary questionnaire was developed based on pertinent regional and international literature, with special consideration given to the limited literature offering guidance for conducting such research in Jordan. The first section of the questionnaire gathered information on the demographic and socio-economic characteristics of the participants (age, education level, employment status, marital status, number of children at home, monthly income, body mass index, pregnancy status, whether they have been diagnosed with diseases during pregnancy, whether they have previous abortion history, whether they think that any of the previous abortion was related to the practice of physical activities, and smoking status). The second section examined physical activities profile for the study participants (whether they practiced moderate physical exercises outside pregnancy during the past year, whether that practiced moderate intensity physical exercises during pregnancy, and in which months of pregnancy have they practiced it). The third section examined participants’ knowledge of safety of practicing specific physical activities regularly during a healthy pregnancy (abdominal exercises, bike riding, swimming, aerobic exercises, walking or jogging, dancing, back exercises, walking, yoga sports, running sports (running), kegel exercises (pelvic exercise), and sports require physical friction like boxing). The fourth section examined participants’ perception, motivations and barriers related to physical activities during pregnancy. In order to interpret participants’ knowledge, the participants were given a score of one for each correct answer, with a maximum score of five for the knowledge scale, the higher the score the more knowledgeable the participants.

### 2.4. Survey validation

The initial survey instrument was pilot-tested on a sample of 39 participants. Piloting of the instrument involved multiple statistical tests to determine its internal consistency, test-retest reliability, and inter-rater reliability because the test was to be administered verbally in an interview protocol.

### 2.5. Statistical analysis

The Statistical Package for Social Science Software, version 29 was used to analyze the data for this study. Continuous variables were presented as mean and standard deviation (SD) for normally distributed variables and as median and interquartile range (IQR) for not normally distributed variables. Categorical variables were presented as frequency and percentage. Predictors of being knowledgeable and practicing moderate physical activity during pregnancy were identified using binary logistic regression analysis. The significance level was assigned as *P* value less than .05.

### 2.6. Patient and public involvement

None.

## 3. Results

### 3.1. Participants’ baseline characteristics

Table 1 below presents participants’ baseline characteristics. A total of 429 participants were involved in this study. More than half of them (60.4%) reported that they hold bachelor’s degree and unemployed or housewife (54.3%). Only 0.7% of them reported that they are divorced or widowed. The median number of children at home was 2.0 (IQR: 1.0–3.0). The median monthly income was 400.0 (IQR: 300.0–600.0) Jordanian dinar. The median body mass index was 28.3 (IQR: 25.7–31.2) kg/cm^2^. Around 17.5% of the study participants reported that they are current smokers. The vast majority of the study participants were current pregnant (87.4%).

**Table 1:** Participants’ baseline characteristics

Variable	Frequency	Percentage
Mean age (SD) years	31.6 (5.9)	
Education level		
Primary school level	19	4.4
Secondary school level	88	20.5
Bachelor’s degree	259	60.4
Diploma	7	1.6
Master degree	56	13.1
Employment status		
Unemployed or housewife	233	54.3
Working in the medical field	82	19.1
Working outside the medical field	105	24.5
Student	9	2.1
Marital status		
Divorced or widowed	3	0.7
Married	426	99.3
Median number of children at home (interquartile range)	2.0 (1.0–3.0)	
Median monthly income (interquartile range) (Jordanian dinar)	400.0 (300.0–600.0)	
Median body mass index (interquartile range) (kg/cm^2^)	28.3 (25.7–31.2)	
Current smoker (yes)	75	17.5
Pregnancy status		
Current pregnant	375	87.4
I was previously pregnant	54	12.6
Have you been diagnosed/had with any of the following during pregnancy?		
Severe anemia (which requires giving iron or blood transfusion)	54	12.6
Early labor (Birth before week 37 of pregnancy)	54	12.6
Bilateral or triple twins	40	9.3
Plastic displacement after 6 months of pregnancy	35	8.2
Cervical surrounding (cervical stitch mode)	18	4.2
Pregnancy poisoning	13	3.0
Membrane rupture	12	2.8
Do you have any previous abortion?		
Yes	166	38.7
Do you think that any of the previous abortion was related to the practice of physical activities? (yes)	52	31.3

SD = standard deviation.

The most commonly reported complication during pregnancy among the study participants were severe anemia (which requires giving iron or blood transfusion) and early labor (Birth before week 37 of pregnancy) accounting for 12.6%. More than one-third of the study participants (38.7%) reported that they have had an abortion before; of which 31.3% think that previous abortion could be related to the practice of physical activities.

### 3.2. Physical activities profile for the study participants

**Figure 1.**
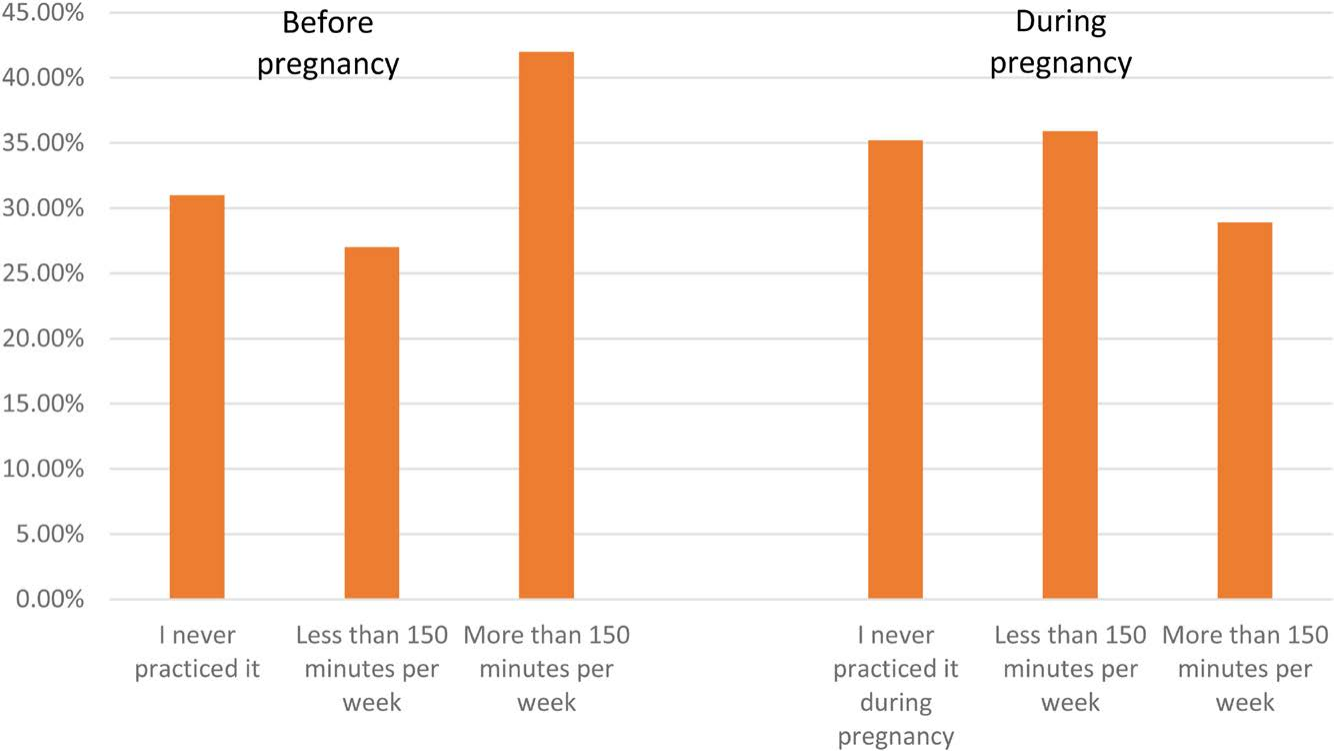
Moderate intensity physical exercises practice before and during pregnancy.

Table 2 below presents physical activities profile for the study participants. Around 69.0% of the study participants reported that they practiced moderate intensity physical activities outside pregnancy during the past year and only 42.0% achieved the recommended duration of 2.5 hours per week. More than half of the study participants (64.8%) reported that they practice moderate physical activity during pregnancy but only 28.9% achieve the recommended duration of 2.5 hours per week. For those who reported that they practice moderate physical activity during pregnancy, more than half of them (59.7%) reported that they practice it during the second trimester during the pregnancy, Figure 1.

**Table 2:** Physical activities profile for the study participants

Variable	Frequency	Percentage
Average or moderate physical activity is known as enough activity to raise your heart rate or\and secrete sweat. Were you doing moderate physical exercises outside pregnancy during the past year?		
I never practiced it	133	31.0
Less than 150 minutes per week	116	27.0
More than 150 minutes per week	180	42.0
Do you practice moderate intensity physical exercises during pregnancy?		
I never practiced it during pregnancy	151	35.2
Less than 150 minutes per week	154	35.9
More than 150 minutes per week	124	28.9
If the answer is yes, in which months of pregnancy did you practice it? (n = 278)(multiple choice question)		
The first trimester during the pregnancy	106	38.1
The second trimester during the pregnancy	166	59.7
The third trimester during the pregnancy	199	71.6

### 3.3. Participants’ knowledge of safety of practicing these physical activities regularly during a healthy pregnancy

**Figure 2.**
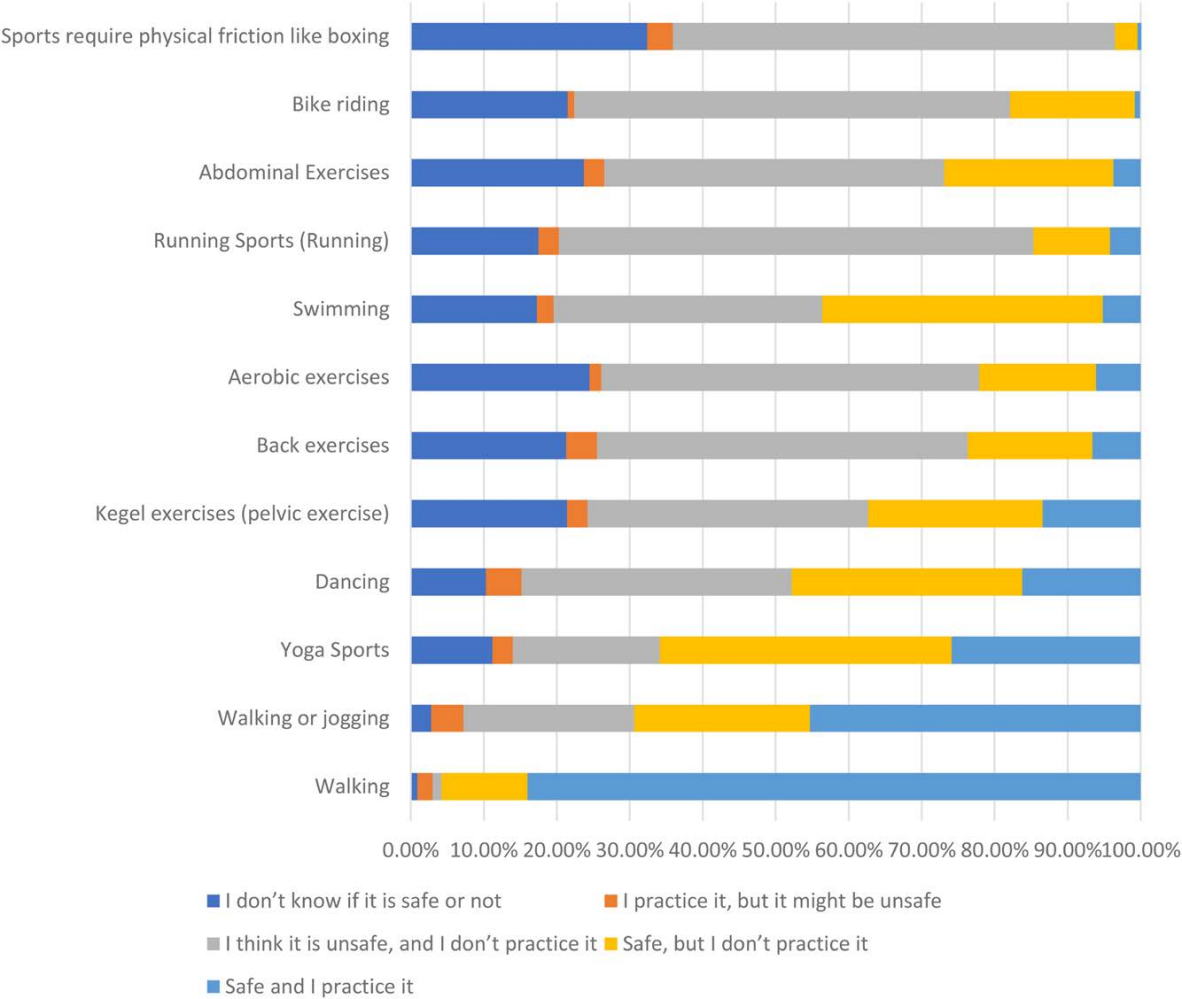
Participants’ knowledge of safety of practicing these physical activities regularly during a healthy pregnancy.

Table 3 below presents participants responses to items that assessed participants’ knowledge of safety of practicing these physical activities regularly during a healthy pregnancy. The most commonly agreed upon type of physical activity for which the study participants knew that it was safe and that they were practicing it was “walking” (84.0%). The least commonly agreed upon type of physical activity for which the study participants knew that it was safe and that they were practicing it was “Sports require physical friction like boxing” (0.5%), Figure 2.

**Table 3:** Participants’ knowledge of safety of practicing these physical activities regularly during a healthy pregnancy

Type of physical activities	I don’t know if it is safe or not	I practice it, but it might be unsafe	I think it is unsafe, and I don’t practice it	Safe, but I don’t practice it	Safe and I practice it
What do you know about the safety of practicing these physical activities regularly during a healthy pregnancy?					
Abdominal Exercises	23.7%	2.8%	46.6%	23.2%	3.7%
Bike riding	21.5%	0.9%	59.7%	17.1%	0.7%
Swimming	17.3%	2.3%	36.8%	38.4%	5.2%
Aerobic exercises	24.5%	1.6%	51.8%	16.0%	6.1%
Walking or jogging	2.8%	4.4%	23.4%	24.1%	45.3%
Dancing	10.3%	4.9%	37.0%	31.6%	16.2%
Back exercises	21.3%	4.2%	50.8%	17.1%	6.6%
Walking	0.9%	2.1%	1.2%	11.8%	84.0%
Yoga Sports	11.2%	2.8%	20.1%	40.0%	25.8%
Running Sports (Running)	17.5%	2.8%	65.0%	10.5%	4.2%
Kegel exercises (pelvic exercise)	21.4%	2.8%	38.5%	23.9%	13.4%
Sports require physical friction like boxing	32.4%	3.5%	60.6%	3.1%	0.5%

### 3.4. Participants’ perception, motivations and barriers related to physical activities during pregnancy

Table 4 below presents participants’ perception, motivations and barriers related to physical activities during pregnancy. The most commonly correctly answered question related to participants’ knowledge of physical activities during pregnancy was that women should not make any effort during pregnancy (97.7%). The least commonly correctly answered question related to participants’ knowledge of physical activities during pregnancy was that exercise helps to maintain weight during pregnancy (73.9%).

**Table 4:** Participants’ perception, motivations and barriers related to physical activities during pregnancy

Variable	Frequency	Percentage
What do you think about the following sentences:		
Women should not make any effort during pregnancy. (False)	419	97.7
Exercise during pregnancy causes miscarriage or premature birth. (False)	412	96.0
It is not recommended to exercise during pregnancy. (False)	408	95.1
Physical exercises are necessary during pregnancy. (True)	399	93.0
Exercise helps to maintain weight during pregnancy. (True)	317	73.9
What encourages you to perform physical activities during pregnancy?		
To relieve stress and tension	286	66.7
To facilitate birth	266	62.0
To relieve back pain associated with pregnancy	212	49.4
Following the advice of the gynecologist and obstetrician	179	41.7
My work requires doing these physical activities	149	34.7
Following the advice of the family doctor or the general doctor	148	34.5
Smartphone applications for exercising at home	123	28.7
What prevents you from performing physical activity during pregnancy?		
Physical exhaustion due to pregnancy	167	38.9
I need relaxation during pregnancy	152	35.4
The doctor did not mention the importance of exercise during pregnancy	124	28.9
I do not know what is the safe sports of a pregnant woman	123	28.7
I am not excited to exercise	111	25.9
I cannot leave my children alone	86	20.0
I did not exercise before pregnancy	81	18.9
I suffered from many abortions	56	13.1
I suffer from an unstable pregnancy (preeclampsia, placenta display, cervical surrounding, pregnancy with twins)	47	11.0
I have a previous negative experience regarding exercise during pregnancy	43	10.0
I suffer from physical disability	20	4.7

The most commonly reported factors that motivate the study participants to perform physical activities during pregnancy were to relieve stress and tension, facilitate birth, and relieve back pain associated with pregnancy, accounting for 66.7%, 62.0%, and 49.4%, respectively. The most commonly reported barriers that prevented the study participants from performing physical activity during pregnancy were physical exhaustion due to pregnancy, need for relaxation during pregnancy, and that the doctor did not mention the importance of exercise during pregnancy, accounting for 38.9%, 35.4%, and 28.9%, respectively.

### 3.5. Predictors of participants’ knowledge and practices

The mean knowledge score among the study participants was 1.8 (SD: 0.8) out of 5 (represents 36.0% of the maximum attainable score); which reflects low level of knowledge of physical activity during pregnancy. Binary logistic regression analysis identified that higher education level and working in the medical field were factors that are associated with higher likelihood of being knowledgeable of physical activities during pregnancy (*P* < .05). On the other hand, being current smokers decreased the likelihood of being knowledgeable of physical activities during pregnancy by 40.0% (*P* < .05). Older participants (aged 31.6 years and above) were 70.0% more likely to practice physical activity during pregnancy compared to others (*P* < .01), Table 5.

**Table 5:** Predictors of participants’ knowledge and practices

Variable	Odds ratio of being knowledgeable (95% confidence interval)	Odds ratio of practicing moderate physical activity during pregnancy (95% confidence interval)		
Mean age categories				
Less than 31.6 years	1.00			
31.6 years and older	1.3 (0.9–2.0)			1.7 (1.1–2.5)**
Education level				
Primary school level	1.00			
Secondary school level	2.8 (1.0–7.5)*	1.4 (0.5–3.6)		
Diploma	5.3 (0.9–32.0)	1.6 (0.6–4.0)		
Bachelor’s degree	4.2 (1.7–10.8)**	0.4 (0.1–2.1)		
Master degree	7.8 (2.5–24.6)***	1.7 (0.6–4.9)		
Employment status				
Unemployed or housewife	1.00			
Working in the medical field	4.8 (2.3–10.0)***	1.4 (0.8–2.4)		
Working outside the medical field	2.0 (1.2–3.4)**	1.6 (1.0–2.6)		
Student	1.2 (0.3–4.8)	5.4 (0.7–43.7)		
Median monthly income categories				
Less than 400 JD	1.00			
400 JD and above	1.4 (0.9–2.2)		1.3 (0.8–1.9)	
Median body mass index categories				
Less than 28.3 kg/cm^2^	1.00			
28.3 kg/cm^2^ and above	1.0 (0.7–1.5)			1.2 (0.8–1.8)
Current smoker				
No	1.00			
Yes	0.6 (0.3–0.9)*	1.4 (0.8–2.4)		
Have you been diagnosed/had with any of the following during pregnancy?				
Severe anemia (which requires giving iron or blood transfusion)	0.9 (0.5–1.7)	1.5 (0.8–2.8)		
Early labor (Birth before week 37 of pregnancy)	0.8 (0.5–1.5)	0.9 (0.5–1.5)		
Bilateral or Triple Twins	1.0 (0.5–2.2)	1.5 (0.7–3.1)		
Plastic displacement after 6 months of pregnancy	0.7 (0.4–1.5)	0.6 (0.3–1.1)		
Cervical surrounding (cervical stitch mode)	0.8 (0.3–2.1)	0.7 (0.3–1.8)		
Pregnancy poisoning	0.6 (0.2–2.0)	0.6 (0.2–1.8)		
Membrane rupture	0.8 (0.2–2.6)	0.5 (0.2–1.7)		
Do you have any previous abortion?				
No	1.00			
Yes	0.9 (0.8–1.1)	0.9 (0.8–1.0)		
Do you think that any of the previous abortion was related to the practice of physical activities?				
No	1.00			
Yes	1.0 (0.5–1.8)	0.8 (0.4–1.4)		

* *P* < .05.

** *P* < .01.

*** *P* < .001.

## 4. Discussion

This study examined knowledge, attitudes, and practices of pregnant Jordanian women towards physical activity during pregnancy. Studying the knowledge, attitudes, and practices of pregnant Jordanian women on exercising during pregnancy is important. It can assist in identifying deficiencies in comprehension and encouraging suitable physical activity, potentially enhancing the health results of both the mother and the fetus.

In our study, around 69.0% of the study participants reported that they practiced moderate physical exercises outside pregnancy during the past year. But only 42.0% achieved the recommended duration of 150 min/week of moderate intensity exercise. A meta-analysis included data from 20 MENA countries showed that the prevalence of physically active adults was 50.8% of adults (ranging from 13.2% in Sudan to 94.9% in Jordan).^[18,19]^ Another study published in 2023 in United Araba Emirates showed that there is low practice of women with about 75.5% has low practice of PA.^[20]^ Besides, in our study we found that more than half of the study participants (64.8%) reported practising moderate physical activity during pregnancy, but only 28.9% achieved the recommended weekly activities of 150 minutes or more. In one study conducted in Campinas, São Paulo, the percentage of adequately exercising women was just over 20% of the women in the study sample. The women in that study were in the third trimester.^[9]^ Another study in United States (US) showed that only 15.8% of pregnant women are achieving the recommended physical activity.^[21]^ For those who reported that they practice physical activity during pregnancy, more than half of them (61.0%) reported that they practice it during the second trimester during the pregnancy. This result was comparable to one study in the United States that showed increased physical activity in pregnant ladies during the second trimester followed by a decline during the third trimester.^[22]^ This result may be attributed to the pregnancy effects on the maternal cardiorespiratory system, which include increases in oxygen consumption, cardiac output, heart rate, stroke volume, and plasma volume. Later on, the increase in oxygen reserve seen in early pregnancy is reduced, leading to maternal exercise, which may present more significant physiologic stress in the third trimester.^[23]^ Also, weight gain and shifting of body gravity lead to progressive lordosis. These changes lead to an increase in the forces across joints and the spine during weight-bearing exercise. As a result, a high percentage of pregnant women experience low back pain with progress of pregnancy.^[24,25]^ Exercise in normal pregnancy is safe in all trimesters. One study conducted in first-trimester pregnant ladies found that exercise decreases the risks of abnormal screening and Gestational Diabetes Mellitus, but the amount needed to decrease the risk is likely higher than current recommendations.^[26]^

According to the ACOG, assuming that women are in good health and experiencing a normal pregnancy, it is considered safe to either continue or initiate regular physical exercise.^[27]^ Engaging in physical exercise does not elevate the likelihood of experiencing a miscarriage, having a baby with low birth weight, or delivering prematurely. In addition, engaging in regular physical activity during pregnancy offers several important advantages for both pregnant women and their developing fetus. These benefits include alleviating back pain, relieving constipation, potentially reducing the likelihood of gestational diabetes, preeclampsia, and the need for cesarean birth, facilitating appropriate weight gain during pregnancy, and enhancing overall fitness while strengthening the cardiovascular system.^[27]^ According to the ACOG, pregnant women should aim to engage in a minimum of 150 minutes of moderate-intensity aerobic activity per week. An aerobic exercise is characterized by the rhythmic movement of major muscles in the body, such as those found in the legs and arms.^[28]^

In our study, we found that the most commonly agreed upon type of physical activity for which the study participants knew that it was safe and that they were practicing it was “walking” (84.0%). This result is the same as other studies that found walking the preferred type of exercise for pregnant ladies.^[9,29]^ Walking is the most chosen type of physical activity during pregnancy, providing several health benefits to both mother and child. Walking also appears to resist many barriers that impede other physical activity modalities during pregnancy.^[18]^

In our study, the most commonly correctly answered question related to participants’ knowledge of physical activities during pregnancy was that women should not make any effort during pregnancy (96.8%). Furthermore, the least commonly correctly answered question related to participants’ knowledge of physical activities during pregnancy was that exercise helps to maintain weight during pregnancy (73.2%). This underscores the necessity for focused educational and awareness initiatives to guarantee that pregnant women are provided with precise information regarding the advantages of engaging in physical exercise while pregnant. Enhancing comprehension in this field could result in improved health results for both mothers and infants, highlighting the need of addressing deficiencies in prenatal care knowledge. As mentioned earlier, there is no significant increase in the risk of miscarriage, low birth weight, or preterm delivery associated with participation in physical exercise. Furthermore, consistent participation in physical activity throughout pregnancy provides numerous significant benefits for both the expectant mother and her developing embryo.^[27]^

In our study, we found that the most commonly reported factors that motivate the study participants to perform physical activities during pregnancy were to relieve stress and tension, facilitate birth, and relieve back pain associated with pregnancy, accounting for 66.1%, 61.4%, and 49.0%, respectively. This is aligning with the exercises benefits reported by the ACOG.^[27]^ Engaging in physical activity while pregnant helps alleviate stress and anxiety by stimulating the production of endorphins, which are the body’s inherent mood-enhancing substances.^[30]^ Additionally, it aids in the preservation of muscle tone and flexibility, so facilitating the process of giving birth. In addition, engaging in regular physical activity can effectively mitigate pregnancy-related back pain by enhancing the strength of the muscles that provide support to the back and enhancing overall posture.^[31]^

In our study, we found that the mean knowledge score among the study participants was 1.8 (SD: 0.8) out of 5 (represents 36.0% of the maximum attainable score); which reflects low level of knowledge of physical activity during pregnancy. Potentially resulting in a dearth of physical activity, this could give rise to fallacies or misunderstandings regarding the safety and advantages of exercising while pregnant. In addition, women’s long-term health may be adversely affected by their inability to embrace healthy behaviors during pregnancy, which could be prevented by a lack of adequate knowledge. Potential complications such as gestational diabetes, preeclampsia, and excessive weight gain may be exacerbated as a result.

In our study, we found that participants who have higher education level and who are working in the medical field were more likely to be knowledgeable of physical activities during pregnancy (*P* < .05). On the other hand, being current smokers decreased the likelihood of being knowledgeable of physical activities during pregnancy by 40.0% (*P* < .05). A chance exists to encourage the mother to engage in healthy behaviors that will benefit her in the short and long term during her pregnancy.^[32]^ In light of the elevated incidence of obesity and cardiometabolic diseases among young women and the general lack of physical activity among this demographic, it is advisable to initiate an educational campaign that promotes the importance of engaging in physical activity while pregnant. As determined by our research, these initiatives ought to be focused on women who are not employed in the medical field and who smoke, as they are less likely to be informed about physical activities during pregnancy.

This study has limitations. The cross-sectional survey study design restricted the ability to examine causality among the study variables. Besides, self-administered survey is prone to social desirability bias, in which the participants might not report their accurate practices and participants may overestimate their activity levels or knowledge to align with perceived social norms. Therefore, our findings should be interpreted carefully.

## 5. Conclusion

In this research, we identified discrepancy in reported physical activity levels, failure to meet the prescribed duration of exercise signifies the necessity for focused interventions. The considerable consciousness surrounding the necessity to practice physical activity while pregnant is a positive development; however, the limited comprehension concerning the contribution of exercise to weight maintenance emphasizes the criticality of education in this regard. The influence of variables including age, smoking status, education level, and occupation on knowledge and practice suggests that distinct subgroups require individualized approaches. Subsequent investigations ought to prioritize the design and implementation of efficacious educational initiatives and interventions aimed at enhancing awareness and encouraging health-conscious physical activity practices throughout pregnancy.

## Acknowledgments

We would like to acknowledge Jordan University of Science and Technology for funding this project.

## Author contributions

**Conceptualization:** Ahlam J. Alhemedi.

**Data curation:** Ahlam J. Alhemedi, Abdallah Y. Naser.

**Formal analysis:** Abdallah Y. Naser.

**Funding acquisition:** Ahlam J. Alhemedi.

**Investigation:** Ahlam J. Alhemedi, Othman Beni Yonis, Nour Abdo, Haya Ali Salem, Esra’a Alomari, Risan Fahmi Alrosan, Qutaiba Alfaqeh, Emran Musadaq Hamza, Abdallah Y. Naser.

**Methodology:** Ahlam J. Alhemedi.

**Project administration:** Ahlam J. Alhemedi.

**Resources:** Ahlam J. Alhemedi.

**Software:** Ahlam J. Alhemedi, Abdallah Y. Naser.

**Supervision:** Ahlam J. Alhemedi.

**Validation:** Ahlam J. Alhemedi, Abdallah Y. Naser.

**Visualization:** Ahlam J. Alhemedi, Abdallah Y. Naser.

**Writing – original draft:** Ahlam J. Alhemedi, Othman Beni Yonis, Nour Abdo, Haya Ali Salem, Esra’a Alomari, Risan Fahmi Alrosan, Qutaiba Alfaqeh, Emran Musadaq Hamza, Abdallah Y. Naser.

**Writing – review & editing:** Ahlam J. Alhemedi, Othman Beni Yonis, Nour Abdo, Haya Ali Salem, Esra’a Alomari, Risan Fahmi Alrosan, Qutaiba Alfaqeh, Emran Musadaq Hamza, Abdallah Y. Naser.
